# Influence mechanism of grinding surface quality of 20CrMnTi steel on contact failure

**DOI:** 10.1038/s41598-024-64320-0

**Published:** 2024-06-11

**Authors:** Long Wang, Lijun Yang, Liuying Wang, Xiujian Tang, Gu Liu

**Affiliations:** 1 Zhijian Laboratory, Rocket Force University of Engineering, Xi’an, 710000 People’s Republic of China; 2 National Defence Key Laboratory for Remanufacturing Technology, Academy of Army Armed Forces, Beijing, 100072 People’s Republic of China

**Keywords:** 20CrMnTi steel, Grinding surface quality, Tribological characteristics, Contact fatigue, Energy infrastructure, Mechanical engineering

## Abstract

To reveal the influence mechanism of the grinding surface quality of 20CrMnTi steel components on the tribological characteristics and contact fatigue performance, accelerated tests for sliding friction wear and fatigue damage were carried out. Tribological characteristics and contact fatigue performance get worse with increasing surface roughness while getting better with increasing surface microhardness. Residual compressive stress is conducive to inhibiting the initiation and propagation of cracks and promoting contact fatigue performance. Additionally, mechanical friction, abrasive wear, adhesive wear and fatigue damage coexist and form a competing failure mechanism under the synergistic effect of frictional wear and contact fatigue failure. The damage process mainly manifests as wear, stress concentration induced fatigue, microcracks, pitting, and spalling in the shallow layer. This study is more beneficial to promote the 20CrMnTi steel transmission parts manufacturing products for high precision, low damage, and long life.

## Introduction

20CrMnTi alloy steel has excellent comprehensive mechanical properties and is suitable for the manufacture of gear, worms, and other transmission parts^1^. It is widely used in automotive, shipping, aerospace, machine tools, and other important fields^2–5^. In practical transmission operations, the surface of 20CrMnTi steel components is subjected to rolling and sliding. Moreover, the failure modes of such surfaces mainly include wear and contact fatigue^6–11^. Various damage phenomena (pitting and spalling) on the machined surface decrease contact accuracy and increase vibration and noise in the transmission process. The tribological characteristics of the component's contact surface affect its working performance, which has attracted extensive attention^12–16^. Thobi et al*.* investigated the tribology and wear aspects of surfaces manufactured by abrasive water jet machining and confirmed the tribological fitness of gears machined at optimum machining parameters^17^. Zhang et al*.* proposed a modeling method to quantify the time-dependent contact parameters of surfaces, which can help in analyzing the time-dependent wear behavior of multi-scale rough surfaces^18^. Zhong et al*.* studied the effects of geometrical characteristics and operating conditions on tribological performance. It demonstrated that the hexagonal textured surface can reduce the friction coefficient compared to the smooth surface^19^. Yao et al*.* investigated the dry sliding friction and wear properties of the stainless steel/ZA8 composite under various temperatures. It pointed out that wear mechanisms are included in delamination wear and plowing wear^20^. Cuao-Moreu et al*.* investigated the wear resistance behavior of the borided surface on the CoCrMo cast alloy under dry-sliding conditions, which increased six times relative to the untreated sample^21^. Yu et al*.* investigated the effect of surface roughness on the bio-tribological behavior of 3D-printed Ti-6Al-4 V alloy samples against Si3N4 counter-balls in a simulated body fluid (SBF) solution. A decreasing trend in surface roughness was correlated with an increase in bio-tribological properties^22^. Xu et al*.* studied the application of electrochemically assisted laser processing for microtexturing aluminum alloy surfaces to enhance their tribological properties. It demonstrated that electrochemically assisted machining reduces surface roughness, allowing textured surfaces to exhibit lower coefficients of friction and less wear under both dry friction and oil lubrication conditions^23^.

Contact fatigue is also an important damage form of the kinematic contact surface^24,25^, which will seriously reduce the transmission accuracy and increase the vibration of the transmission process. Prieto et al*.*^26^ analyzed the rolling contact fatigue resistance of cryogenically treated AISI 440C steel. Although cryogenic treatment was beneficial to reduce the number of weak interfacial regions at the microstructure level, it did not significantly improve contact fatigue resistance. Liu et al*.*^27^ pointed out that the contact fatigue estimation of carburized turbine gear was influenced by the combination of mechanical properties, lubrication, and load spectrum. Mostafa et al*.*^28^ studied the microstructural alterations of SAE 52,100 bearings related to the rolling contact fatigue processes. The deterioration thickness of the fatigue damage region increased with contact pressure and stress cycles. Everitt et al*.*^29^ analyzed the influence mechanisms of asperities and indents on contact fatigue characteristics by combining experimental research with numerical simulation. The contact fatigue crack was caused by the thermal elastohydrodynamic lubrication load cycle, and the pitting was caused by the tensile residual stresses from plastic deformation. Lai et al*.*^30^ investigated the mechanism of crack formation in the bearing premature fatigue failure process. Tensile stress drove the early initiation and accelerated growth of cracks from the pre-existing defects, leading to the occurrence of premature failure. Faruk et al*.*^31^ studied the impact of cryogenic grinding on the surface integrity and fatigue performance of carburised 27MnCr5 steel. It indicated that cryogenic grinding produces surfaces with minor roughness and compressive residual stresses that are 10–20% lower than those generated by conventional wet grinding. Wu et al*.*^32^ analyzed the effects of Ultrasonic Surface Rolling Process (USRP) on the surface integrity and rolling contact fatigue behavior of 18CrNiMo7-6 steel. USRP significantly refined the surface to a remarkably smooth finish and introduced a substantial layer of residual compressive stress, which led to an enhanced rolling contact fatigue life.

Friction is an important factor leading to the mechanism of gear failure, while strength measures the ability of a structure to resist failure, and interface contact is the bridge connecting the two. The frictional characteristics, contact fatigue characteristics, and life prediction of gear surfaces are important research directions in the field of mechanical engineering. Through comprehensive research on the surface integrity, contact characteristics analysis, gear surface damage mechanism, and service life analysis of gear machining, the working performance of gears can be effectively improved. However, there are fewer studies on the coupled synergies of surface integrity, tribological characteristics and contact fatigue performance. Through reciprocating sliding friction and wear tests and rolling contact fatigue tests, the influence mechanism and law of grinding surface integrity on tribological characteristics and contact fatigue performance were deeply explored. Therefore, the wear mechanism of the surface integrity and lubrication state of the grinding surface on the tribological characteristics were investigated using reciprocating sliding friction and wear tests. Meanwhile, by carrying out rolling contact fatigue tests on the end faces of 20CrMnTi workpieces, the influence mechanism and law of grinding surface integrity on contact fatigue performance were deeply explored. It can provide important experimental and theoretical bases revealing the influence mechanism of the machined surface quality on the service performance of 20CrMnTi steel components and is beneficial to optimize the manufacturing level and promote the transmission parts to high precision, high transmission efficiency, low noise, and long life.

## Experimental conditions and methods

The 20CrMnTi carburized and quenched steel ring workpiece has dimensions of *Φ* 60 mm × 6 mm × *Φ* 30 mm. The carburized layer depth of 20CrMnTi alloy steel is about 1.1 ~ 1.3 mm, the surface hardness is 59 ~ 64 HRC, and the core hardness is 36 ~ 42 HRC. The working surface was machined using the microcrystal corundum grinding wheels. Shot peening was carried out to improve the residual stress on the machined surface. The machined surface roughness was measured by using the LINKS 2300A-RC profile roughness measuring instrument. The surface hardness was measured by the MICROMET-6030 automatic microhardness tester of the American Standard Lok Company. The surface residual stress was measured by the Xstress 3000 stress analyzer.

**Figure 1:**
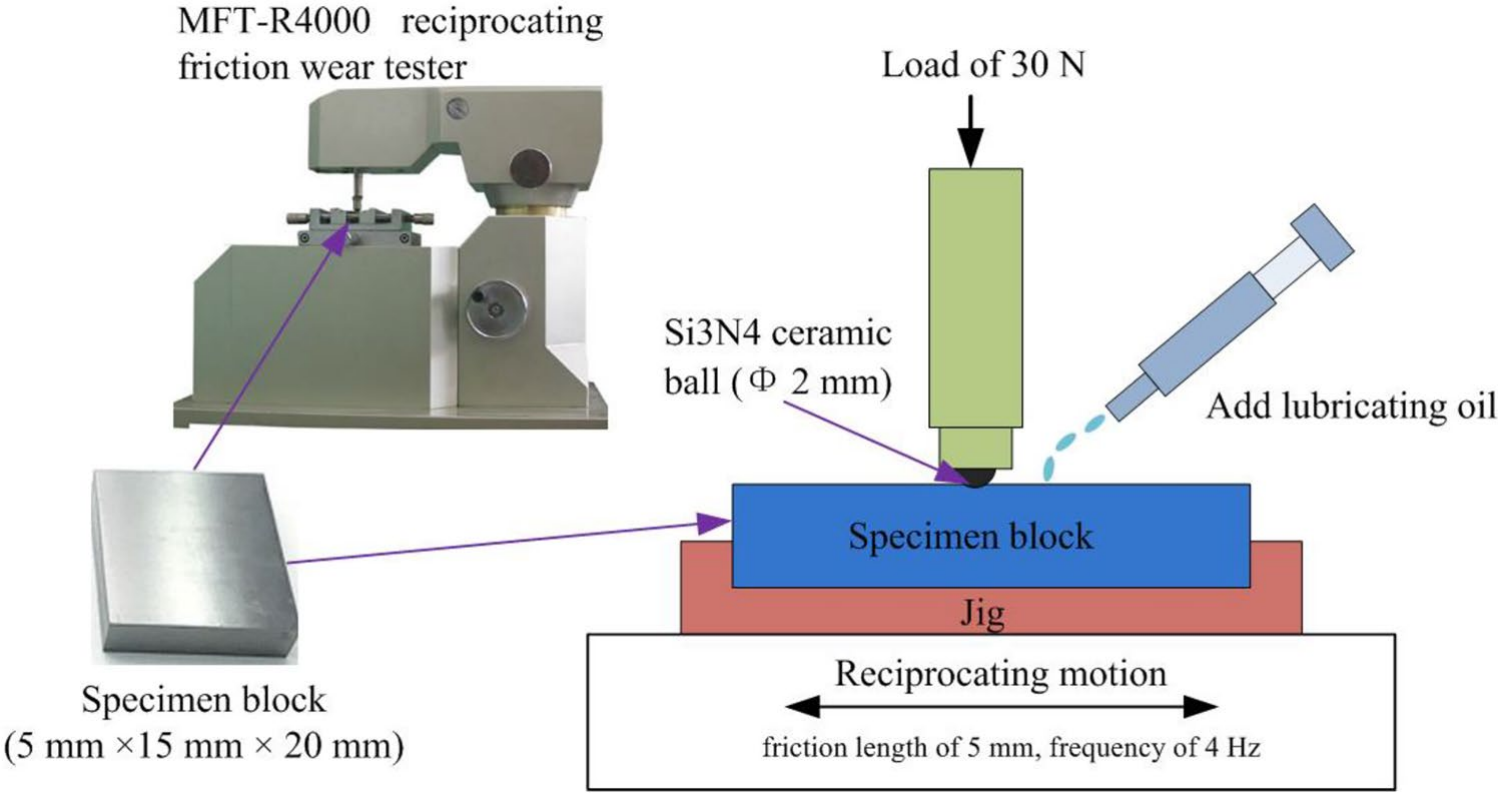
Reciprocating sliding friction and wear test.

As shown in Fig. 1, The carburized and quenched 20CrMnTi alloy steel was cut into workpiece blocks with dimensions of 5 mm × 15 mm × 20 mm, and the surface (size of 15 mm × 20 mm) was machined by grinding. Reciprocating sliding friction and wear tests were conducted using the MFT-R4000 tester (manufacturer: Lanzhou Institute of Chemical Physics, Chinese Academy of Sciences). The main indexes of the MFT-R4000 reciprocating friction wear tester were: loading range was 10 ~ 200 N, reciprocating frequency was 0.1 ~ 25 Hz, and reciprocating length was 5 ~ 40 mm. Reciprocating friction motion was conducted on the machined surface by employing a silicon nitride ceramic ball (*Φ* 2 mm) for 20 min at an amplitude of 5 mm, a frequency of 4 Hz, a normal load of 30 N under the applied voltage of 1.5 V. The temperature and relative humidity were 25 ℃ and 30%. The machined surface is in dry sliding contact or under hydrodynamic lubrication. The yellow-shell HX5 mineral lubricating oil was used as the hydrodynamic lubricant.

**Figure 2:**
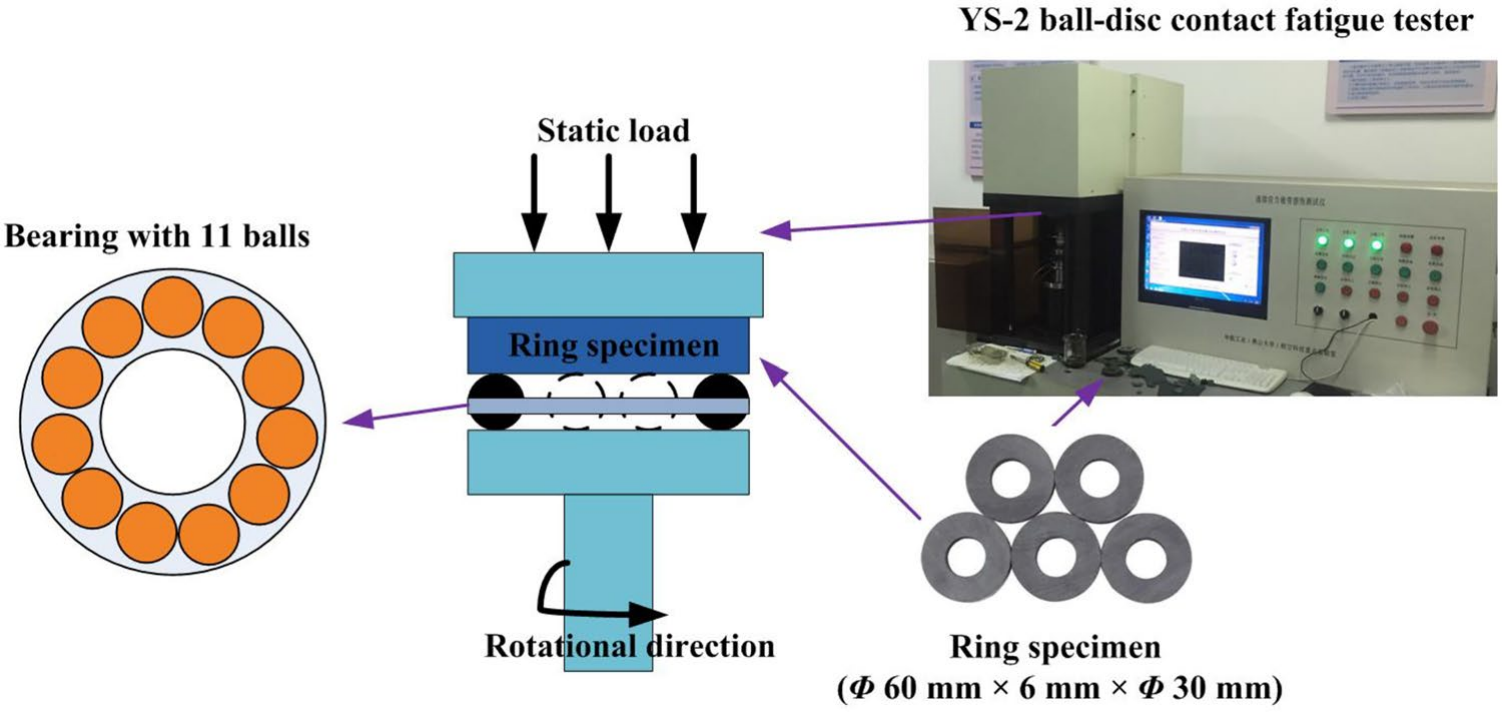
Rolling contact fatigue test.

As shown in Fig. 2, the rolling contact fatigue test on the workpiece surface was carried out by applying the YS-2 ball-disc contact fatigue tester for fatigue damage assessment under continuous stress. The bearings consist of 11 GCr15 steel balls (*Φ* 5.56 mm) and were used to form friction pairs with the ring workpiece. The rotation rate of the tester was set to 4000 rpm, while the applied normal stresses were 2500 to 3500 MPa. Due to the machined surface material removal caused by fatigue, the bearing will produce strong vibration under high-speed operation. The YS-2 ball-disc contact fatigue tester can monitor vibration signals online in real-time. When the amplitude of the vibration signal exceeds the threshold value (5 g) for 30 consecutive times, fatigue failure can be considered to have occurred, and the tester can automatically be shut down and record the number of revolutions^33^.

## Results and discussion

### Analysis of tribological characteristics

#### Analysis of frictional wear morphology

**Figure 3:**
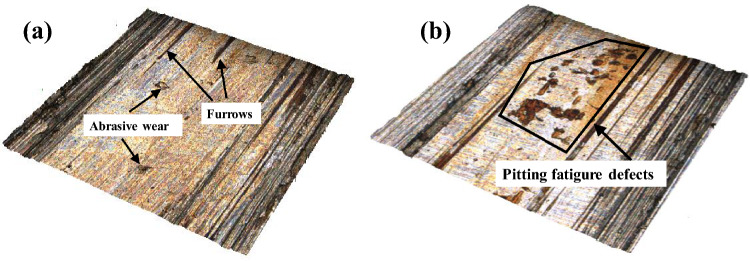
Wear morphology of dry sliding friction: (**a**) Light wear morphology; (**b**) Severe wear morphology.

The micromorphology of the worn surface in dry sliding contact is shown in Fig. 3. It can be seen from Fig. 3a that the worn surface mainly shows significant abrasive wear and furrows induced by scratching. Under the effect of reciprocating friction, a scratch whose inclination angle was parallel to the friction direction appeared on the contact surface. In the frictional wear process, the morphology of the contact surface was constantly planished owing to numerous asperities being affected by plastic deformation and micro-cutting. As shown in Fig. 3b, surface pitting fatigue defects of different sizes appeared on the workpiece surface, and the contact fatigue damage aggravated the frictional wear of the contact surface. Numerous imperfections on the rough surface of the workpieces were transformed into different sizes of micro-pitting defects due to the micro-spalling of materials caused by cyclically accumulated plastic deformation or local stress concentration. The sharp part of the micro-etched pits was successively subjected to a stress concentration effect, which caused the nucleation and propagation of micro-cracks to result in macroscopic spalling.

**Figure 4:**
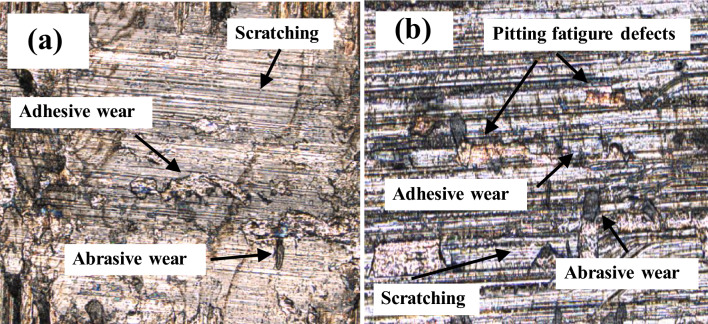
Surface wear morphology in lubrication state: (**a**) Light wear morphology; (**b**) Severe wear morphology.

In the lubrication state, the wear morphology of the workpiece surface is displayed in Fig. 4. As shown in Fig. 4a, the worn surface of the workpiece presented distinct ploughed furrows and plastic deformation, as well as significant adhesive wear induced by material transmission. The wear modes of the workpiece surface were scratching, adhesive wear, and abrasive wear. Owing to the friction process occurring in a mixed lubrication state, many asperities were affected by significant extrusion and reciprocating friction effects. Therefore, the metal materials underwent plastic deformation flow and adhered to the contact surface. Additionally, when the internal high-shear stress of asperity materials reached its ultimate strength, the surface micromorphology was constantly planished to form hard debris. Afterwards, the hard debris was mixed into the lubricant or adhered to the workpiece surface to cause abrasive wear. It can be seen from Fig. 4b that, when the workpiece surface was heavily worn, ploughed furrows of different depths whose inclination angles were parallel to the slip direction appeared on the contact surface. Additionally, there were numerous scattered pits caused by fatigue pitting on the surface, and in this context, the wear mechanism of the workpiece was shown as the coupled effect of friction wear and fatigue damage. When the plastic deformation of the surface accumulated to a certain degree, stress concentration occurred due to the presence of some defects, such as furrow marks, that formed micro-cracks. With the number and propagation length of initiating cracks gradually increasing, fatigue pitting was formed, causing the materials to spall and form hard debris. Furthermore, the hard debris participated in the micro-cutting action and intensified the friction and wear of the workpiece surface.

#### Effect of grinding surface quality on tribological characteristics

**Figure 5:**
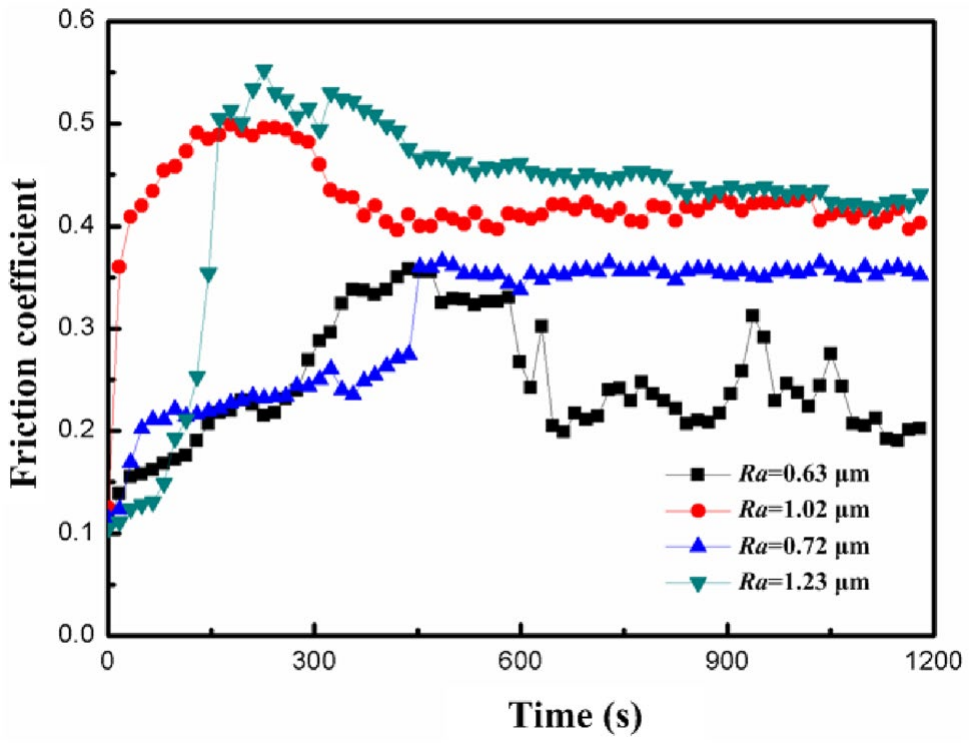
Effect of surface roughness on the dry friction.

Surface roughness is an important index used to describe the microcosmic unevenness of the workpiece surface. In the process of dry sliding wear, the change in friction coefficients of surface morphologies with varying roughness over time is shown in Fig. 5. The whole wear process was composed of running-in and steady-state wear. The friction coefficients increased in the initial rapid running-in phase: the convex tip of the surface morphology came into contact with a small initial contact area at first, which rapidly removed the grinding trace in the contact area. Moreover, the friction coefficient reached a steady state in the steady wear phase. When numerous convex peaks in the workpiece surface gradually underwent significant plastic deformation, the surface roughness also gradually decreased while the total contact area gradually increased. Moreover, the bearing capacity and wear resistance increased, and the friction coefficient gradually tended to a stable value. The larger the surface roughness *R*_*a*_, the larger the friction coefficient. This was because the larger the surface roughness, the larger the compaction height of the micro-convex peaks on each contact surface and the larger the contact stress and plastic deformation, which aggravated the adhesive wear. Additionally, increasing surface roughness led to an obvious stress concentration in the sharp valleys of the surface profile, which promoted the initiation of near-surface contact fatigue cracks. On this basis, the contact strength worsened to exacerbate the frictional wear.

**Figure 6:**
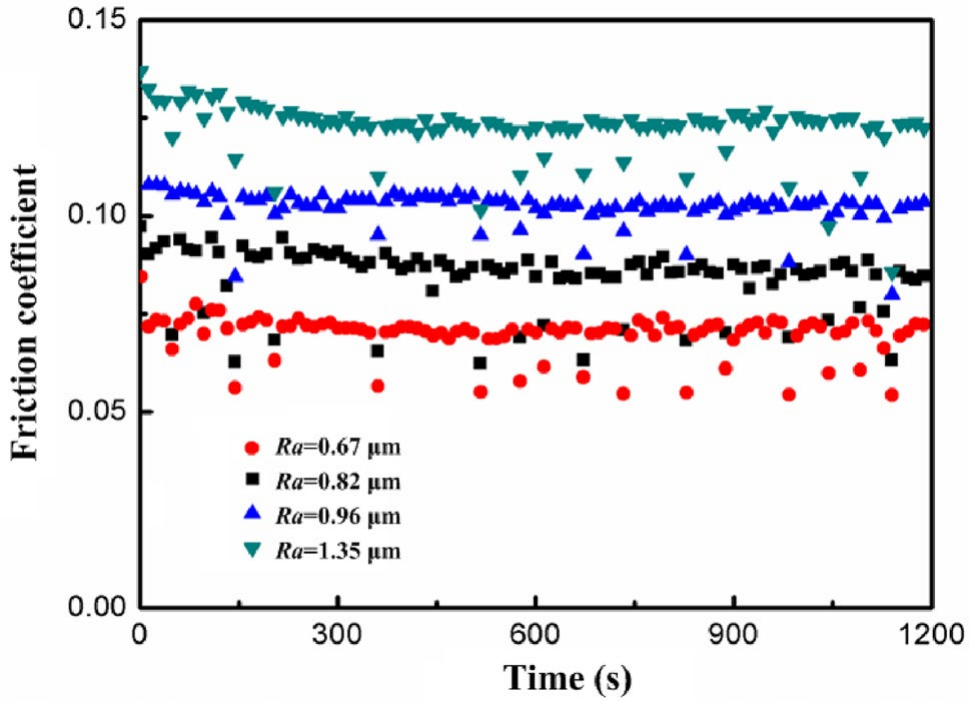
Effect of surface roughness on the lubrication friction.

Changes in the wet friction coefficient of different rough surfaces are separately shown in Fig. 6. Due to the effects of hydrodynamic lubrication, the friction coefficient can retain a steady state value, which significantly changes the tribological characteristics of the workpiece surfaces. Compared with the dry sliding process, the friction coefficient decreased to realize the associated friction-reduction and wear-resistance effects. Due to the relative motion between the surfaces of the friction pairs, the viscous hydrodynamic lubricant film was conveyed into the gaps between numerous peaks and valleys on the workpiece surface profile to induce a hydrodynamic effect. This bore part of the external loads and reduced the friction wear. Additionally, the friction coefficient increased with increasing surface roughness. When the surface roughness was low, the thickness of the oil film exceeded the height of the asperities on the contact surface to cover the uneven nature thereof. In this context, the friction testing was conducted in an elastic hydrodynamic lubrication state. The more significant the unevenness of the surface profile, the more the asperities undergo direct contact, which results in a mixed lubrication state. The hydrodynamic lubrication effect was gradually weakened while the boundary lubrication effect remained significant, resulting in a gradual increase in the friction coefficient. The synergistic effect between surface roughness and lubricating oil films affected the tribological characteristics of such lubricated contact.

Tallian et al*.* proposed the concept of oil film thickness ratio *λ* determined by the combined surface roughness *R*_*a*_ of the friction pair and the elastic–plastic oil film thickness *h*_min_. When the oil film thickness ratio *λ* > 3, the thicker oil film avoids localized micro-contact details and functions in a dynamic pressure lubrication state with minimal wear. When the oil film thickness ratio 1 < *λ* < 3, the system operates in a mixed lubrication state where part of the oil film completely separates the friction surface, while other areas experience micro-convex body contact in a boundary lubrication state, leading to further wear. When the oil film thickness ratio *λ* < 1, the system is in a boundary lubrication state, resulting in significantly reduced lubricant fluid effectiveness and increased wear. If *R*_*a*1_ represents the surface roughness of 20CrMnTi workpiece, R_*a*2_ denotes the surface roughness of silicon nitride grinding ball, *h*_min_ is the minimum lubrication film thickness between the surfaces of friction sub-surfaces, the oil film thickness ratio *λ* is expressed as Eq. (1). It was evident that increasing tooth surface roughness weakens the dynamic pressure lubrication effect and enhances the boundary lubrication effect, consequently leading to higher coefficients of friction and increased wear levels.

1 $$ \lambda = h_{\min } \left( {R_{a1}^{2} + R_{a2}^{2} } \right)^{ - 0.5} $$

**Figure 7:**
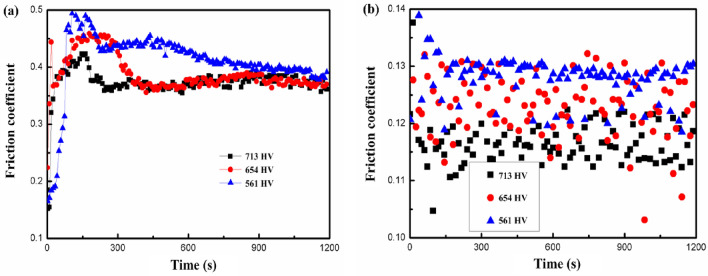
Relationship between friction coefficient and microhardness: (**a**) Dry friction; (**b**) Lubrication friction.

When the machined surface roughness of 20CrMnTi steel was 0.87 μm, the sliding friction and wear test was conducted on the workpiece surfaces at different microhardnesses (separately) in dry friction and hydrodynamic lubrication conditions. It can be seen from Fig. 7 that the friction coefficients all decreased with increasing microhardness, while the friction-reducing effect was weaker than the surface roughness. In terms of the value of the friction coefficient in a lubricated state, the friction process was conducted mainly in a mixed lubrication state (mainly manifested as boundary lubrication). It was essential to take the influence of the elastic deformation and pressure of contact areas on the viscosity of the lubricant into account. The microhardness of materials exhibited a positive correlation with the elastic modulus^34^: the larger the microhardness, the larger the elastic modulus, and the higher the fatigue resistance of the workpiece surface, which was conducive to improving the wear resistance of such materials. The improvement in microhardness of the workpiece surfaces can reduce the rate of frictional wear thereof and is also conducive to improving the adhesive wear condition of surfaces, thus further decreasing the friction coefficient.

When lubricating the contact state, it is even more essential to consider the elastic deformation of the contact zone surface and the impact of pressure on the lubricant's viscosity. If *α* represents the viscous pressure coefficient, *η* denotes the dynamic viscosity, *v* stands for the relative sliding speed, *R* indicates the equivalent radius of curvature, *E* represents the equivalent modulus of elasticity, and *P*_n_ is the normal load per unit of contact length. The empirical formula derived by Dawson et al*.* in the UK for calculating the minimum oil film thickness, *h*_min_, required to establish an elastic-fluid lubrication state is expressed in Eq. (2). It was evident that as the modulus of elasticity and normal load decrease, a smaller oil film thickness was needed to ensure the formation of the dynamic pressure lubrication state, making it more likely to occur. Furthermore, it can be observed that an increase in the microhardness of the tooth surface, tantamount to enhancing the modulus of elasticity, promotes the formation of the dynamic pressure lubrication state, thereby achieving improved friction and wear resistance effects.

2 $$ h_{\min } = 2.65\alpha^{0.54} \eta^{0.7} v^{0.7} R^{0.43} E^{ - 0.03} P_{n}^{ - 0.13} $$

In summary, there were significant differences in the curve shape and numerical values of the friction coefficient between dry friction and lubrication friction. Firstly, the friction coefficient of wet friction was much lower than that of dry friction. Due to the formation of a lubricating layer on the contact surface, the magnitude of friction was reduced, making the operation of the contact surface smoother and more efficient. Secondly, due to the lack of lubrication layer protection between the contact surfaces during dry friction, the small protrusions on the surface would collide with each other during the friction process, leading to rapid wear, fatigue, and chip shedding of the material during the running-in period, resulting in the friction coefficient curve showing two stages: running-in wear and steady-state wear.

### Analysis of contact fatigue performance

#### Morphology analysis under contact fatigue damage

**Figure 8:**
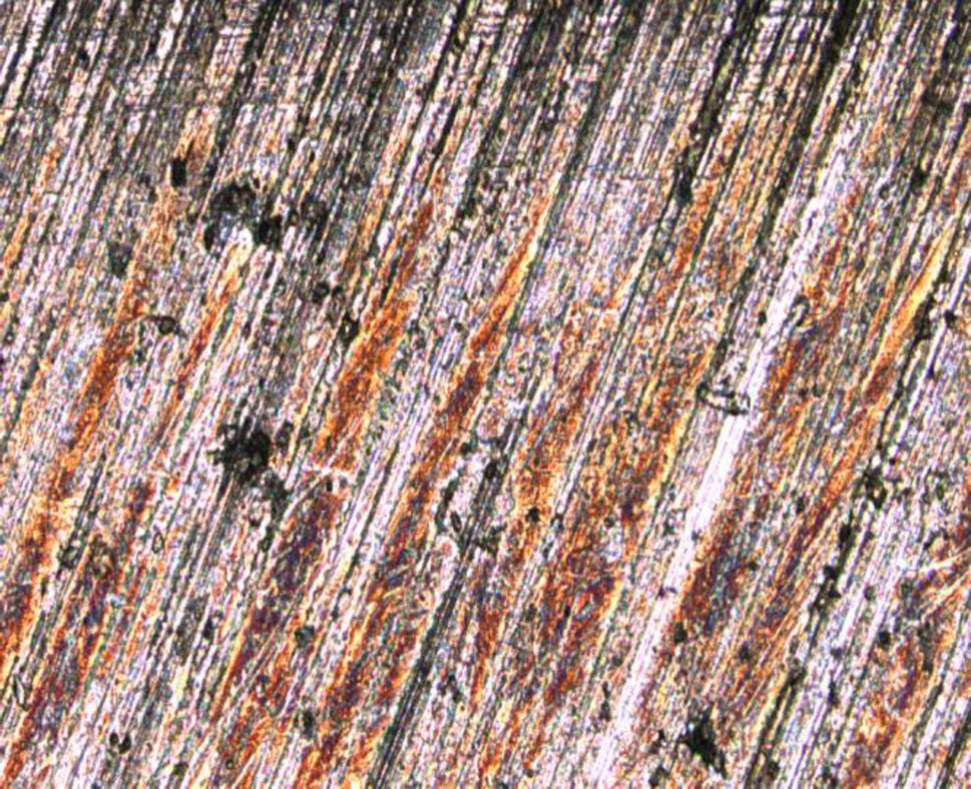
Initial wear morphology of contact fatigue damage.

Figure 8 shows that the friction pairs were in intimate contact under the reciprocating friction motion in the contact ring, causing the rough surface to be eliminated. When the asperities on the rough surface made contact with the ball-bearings in rolling mode, the asperities were subjected to significant plastic deformation and caused the adhesive wear in the rolling contact area to produce a large shear stress. The asperities on the machined surface were constantly removed under the synergistic effects of extrusion and shear. Fatigue pitting with a swallow depth (20 to 30 μm) formed within the wear scar on the workpiece surface. In rolling modes of contact, there were significant wear phenomena observed to have further caused contact fatigue. Actually, contact fatigue can be regarded as an important form of material wear. Contact fatigue appears stochastically as pitting, spalling, and defects on the contact surface.

**Figure 9:**
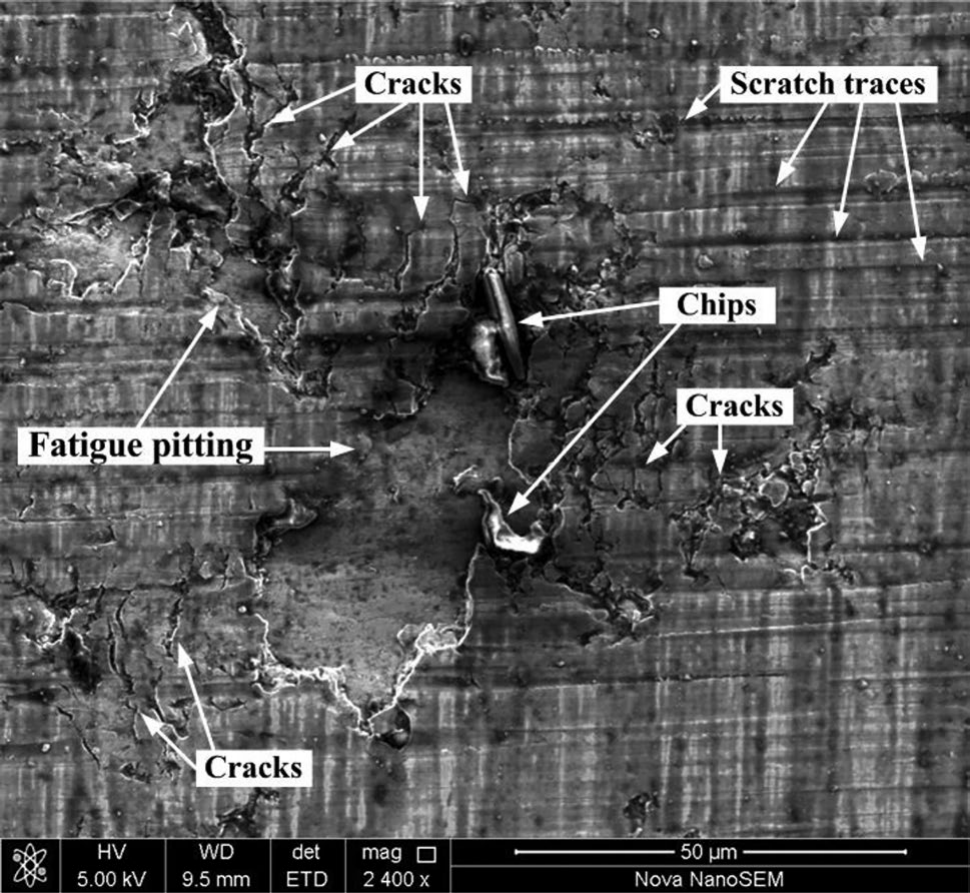
Micromorphology of pitting.

The micromorphology of the fatigue pitting is shown in Fig. 9. The damaged surface morphology was characterized by abrasive wear, adhesive wear, fatigue damage, scratching, and plastic deformation. The excessive surface asperities on the surface folded after upheaval of the micromorphology of the surface under compressive stress. The local Hertz contact stresses manifested in the folding sites of asperities were far larger than the macroscopic Hertz contact stresses. The deformation folding and local stress concentrations led to the formation of microcracks, which propagated along the direction parallel to the contact surface to form pitting. The diverse micro-defects near the surface, including grinding marks and wear scars, are important precursors to fatigue pitting.

**Figure 10:**
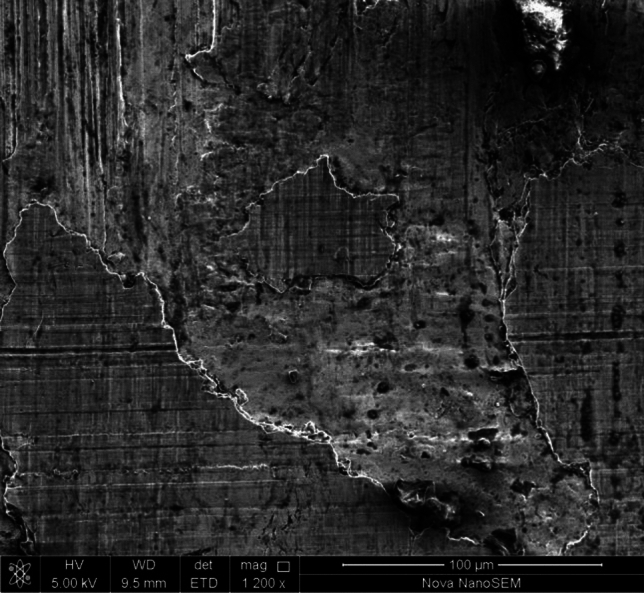
Micro morphology of fatigue spalling.

As shown in Fig. 10, fatigue spalling was characterized by a large area and a shallow depth. Fatigue spalling was probably caused by the combined action of maximum orthogonal stress and maximum shear stress. The stress concentration at the edge of micro-pitting pits would further induce the number and propagation length of micro-cracks to increase rapidly, and the damage area and depth of local areas to increase continuously. Meanwhile, microcracks mainly expand and connect rapidly along the same plane until the formation of spatial closure, prompting the occurrence of a large area of spalling in the shallow layer.

#### Effect of grinding surface quality on contact fatigue performance

**Figure 11:**
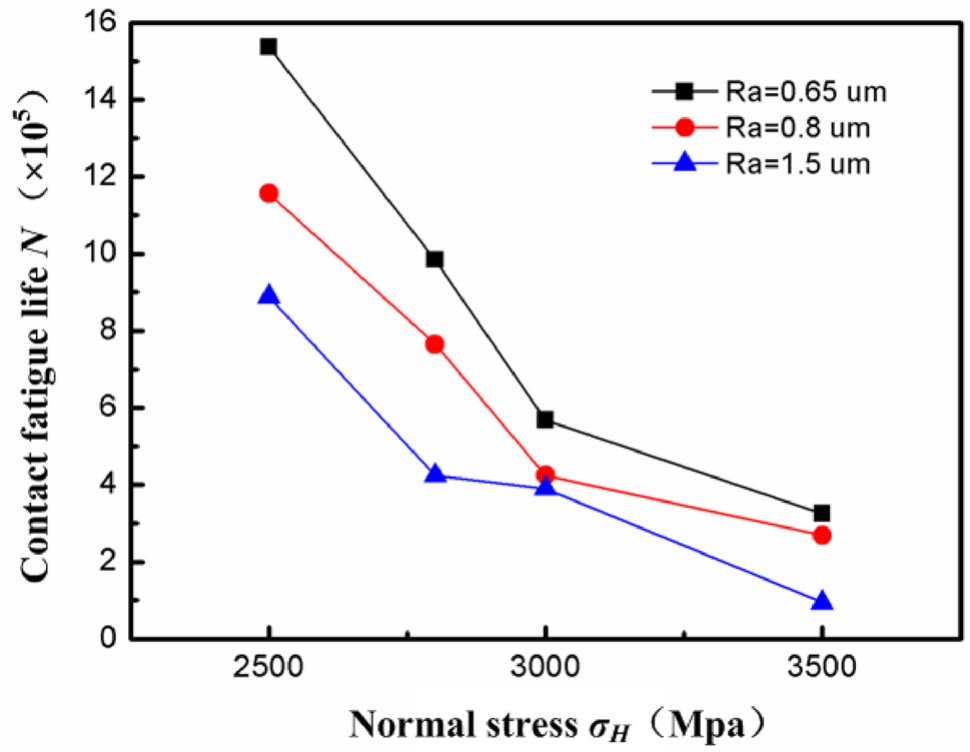
Influence of surface roughness on contact fatigue life.

Surface roughness exerts an important effect on contact fatigue performance. By investigating the influences of three surface roughnesses (0.65, 0.8, and 1.5 μm) on the contact fatigue performance. As shown in Fig. 11, with increasing surface roughness *R*_*a*_ and normal stress *σH*, the contact fatigue life *N* of the workpiece decreased. Under low normal stress, the surface roughness had a greater influence on the contact fatigue life. The fatigue life of the workpiece can improve by 1.5 to 2 times when the surface roughness *R*_*a*_ decreases from 1.5 μm to 0.65 μm. There were three reasons for this: firstly, the low roughness was conducive to reducing the stress concentration caused by local asperities and preventing the initiation of contact fatigue cracks near the surface. Secondly, the lower the roughness, the smoother the surface, which can reduce friction and wear, reduce thermal damage caused by friction, and thus extend its service life. Thirdly, the low roughness was conducive to decreasing direct contact between asperities on the surface, thus otherwise weakening the boundary lubrication effect and strengthening the dynamic lubrication effect. In summary, in engineering design, it is necessary to strictly control the surface roughness of parts to improve their fatigue life and safety of use.

**Figure 12:**
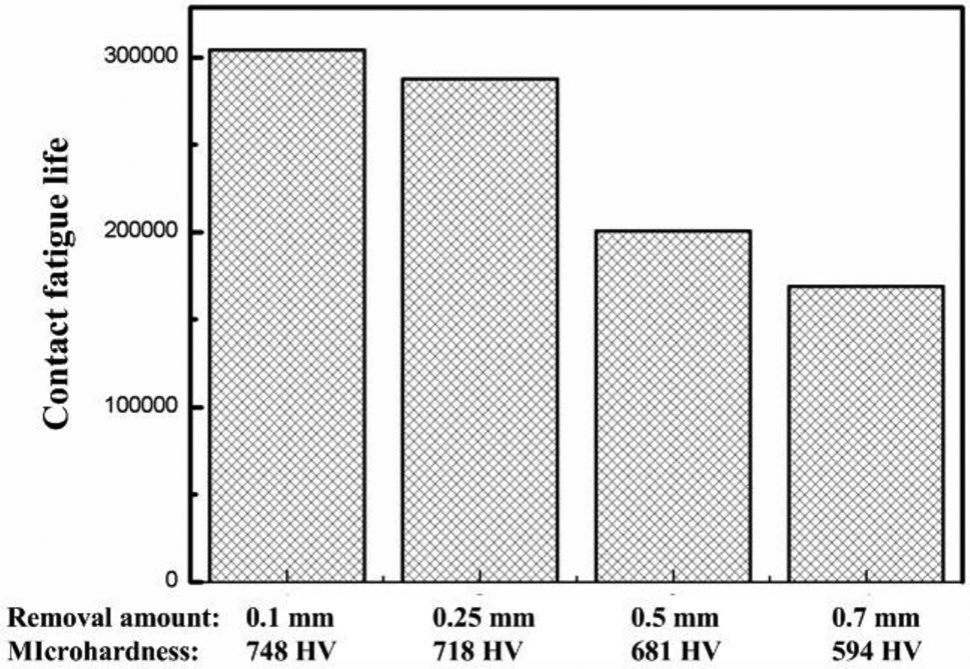
Influence of microhardness on the contact fatigue life.

Due to removing different thicknesses of materials, different depths of carburized layers can be held, and the surface roughness was guaranteed to be 0.72 μm. The experiment on the influence of holding different depths of effective hardening layers on contact fatigue performance was carried out in which the applied normal stress was 3500 MPa. The influence of microhardness on contact fatigue life is shown in Fig. 12. The larger the amount of grinding removal, the lower the microhardness of the machined surface. Moreover, the contact fatigue life of the workpiece was reduced. Because surfaces with lower hardness are more difficult to resist local high stress and wear, which increases surface damage and makes it more difficult to maintain the stability of the contact area. Meanwhile, the decrease in surface hardness also makes it more difficult to maintain surface flatness and roughness, further reducing the contact quality of the surface. In summary, The surface microhardness was positively correlated with the elastic modulus^35^, which was conducive to increasing the plastic deformation resistance and shear strength of such materials. These characteristics enable surfaces with higher hardness to have a longer fatigue life.

**Figure 13:**
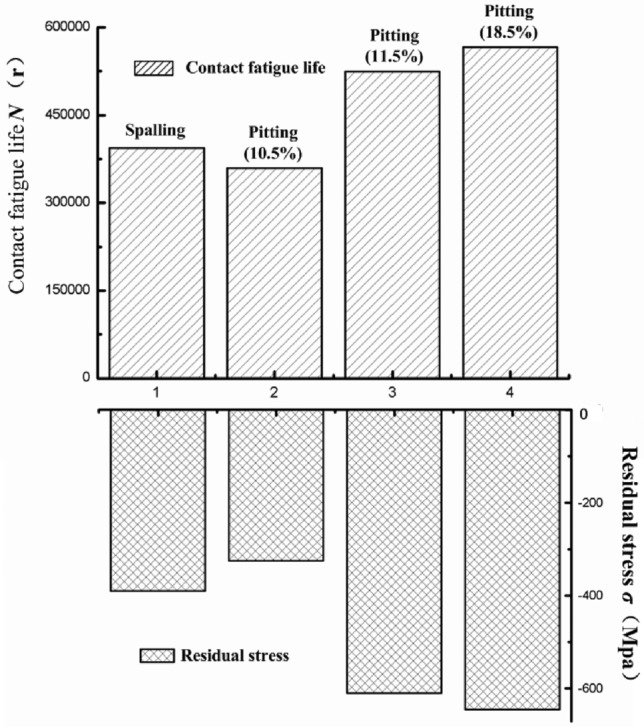
Influence of residual stress on the contact fatigue life.

To investigate the influence of residual stress on contact fatigue performance, experiments undertaken before and after shot peening were analyzed: Samples 1 and 2 were only subjected to carburizing and quenching treatment; samples 3 and 4 underwent carburizing, quenching, and shot peening. The rate of rotation and applied normal stress were separately set to 4000 rpm and 3000 MPa, and the test results are as shown in Fig. 13. After shot peening, the residual compressive stress on the surface layers of the workpieces increased significantly, and the contact fatigue life was much improved. As shown by the failure mode, the workpieces treated with strengthened shot peening mainly presented pitting and wear to their surfaces, while those not subjected to shot peening might show light spalling. Above all, the use of strengthened shot peening was favorable to increasing the residual compressive stress in the surface layers, enhancing contact fatigue strength, and prolonging the contact fatigue life. The reason for these benefits was that the larger residual compressive stress helps to increase the closing force across microcracks to slow the rate of crack propagation.

## Conclusions

The friction coefficient increases with increasing surface roughness while decreasing with increasing surface microhardness. The smaller the surface roughness, the more conducive it is to reducing the concentration of contact stress under certain surface morphology characteristics and even to improving the hydrodynamic lubrication effect in the lubrication friction process. The higher the surface microhardness, the more effectively the elastic modulus is increased, thereby improving the adhesive wear conditions. The smaller the surface roughness and the larger the microhardness, the better the fatigue damage resistance of the material. In addition, shot peening can significantly increase the residual compressive stress on the surface layer of the workpiece and suppress the initiation and propagation of contact fatigue cracks.

Friction wear and contact fatigue failure coexist, forming a competitive failure mechanism. The damage process mainly manifests as wear, stress concentration induced fatigue, microcracks, pitting, and spalling. The synergistic effect of surface morphology, hardness, roughness, and residual stress on surface integrity has a complex, comprehensive impact on tribological performance and contact fatigue performance. However, this study is mainly based on single-factor research and has not considered the mechanism of multi-factor coupling. In the future, artificial intelligence algorithms can be used to analyze the tribological and fatigue performance of multiple inputs and multiple outputs, promoting the selection of better surface integrity, thereby improving interface conditions and extending service life.

## Data Availability

The authors confirm that the data supporting the findings of this study are available within the article.
